# Medicinal Plant Extracts Evaluated *In Vitro* and *In Vivo* for Antidiabetic Activities in Ethiopia: Bases for Future Clinical Trials and Related Investigations

**DOI:** 10.1155/2021/9108499

**Published:** 2021-09-04

**Authors:** Berhan Begashaw Yikna, Awgichew Shewasinad Yehualashet

**Affiliations:** Pharmacology and Toxicology Unit, Department of Pharmacy, College of Health Sciences, Debre Berhan University, Debre Berhan, Ethiopia

## Abstract

**Background:**

Diabetes mellitus (DM) is a metabolic disorder characterized by a persistent rise in the blood glucose level resulting from defects in cellular insulin function, secretion, or both, which affects millions of people every year. Several drawbacks have been stated with the use of marketed antidiabetic medicines such as drug resistance, adverse effects, toxicities, and even costs. Due to these several limitations, searching for novel antidiabetic medicines from medicinal plants (MPs) is becoming an active area of research. Therefore, MPs are exemplary sources of medicines with many accessible agents being obtained from them because numerous active constituents are isolated from them for direct use as pharmacological medicines or act as lead compounds. This paper was aimed to synthesize a concluding remark using *in vitro* and *in vivo* evaluations of extracts and fractions for antidiabetic potentials in Ethiopia, which can be used to direct future clinical trials and related investigations.

**Method:**

So as to get data on the different investigations, publications related to experimental evaluations on animal diabetic models in Ethiopia were searched from databases, such as Google Scholar, Web of Science, Medline, PubMed, and Scopus using English key terms.

**Results:**

In this paper, about 37 research findings based on data from various areas of Ethiopia published until the end of November 2020 were included. A total of 37 MP species extracts and fractions belonging to 19 families have been revealed *in vitro* or *in vivo* for potential antidiabetic activities. Crude extracts were carried out mostly by hydromethanolic whereas fractions were done mostly by chloroform. Leaves were the most commonly experimentally investigated plant part. Among the MP species experimentally studied, the most frequently used to treat DM in Ethiopia were *Thymus schimperi* Ronniger (Lamiaceae), *Moringa stenopetala* (Baker f.; Moringaceae), *Ajuga remota* Benth (Lamiaceae), and *Datura stramonium* Linn. (Solanaceae).

**Conclusion:**

This paper gives aggregate evidences on the potential antidiabetic activities of MPs in Ethiopia. Antidiabetic MPs used in Ethiopia represent crucial input for the future development of novel antidiabetic drugs. To this end, more pharmacological and toxicological investigations need to be considered to prove the safety of constituents obtained from these MPs. Finally, we recommend upcoming research to ensure future success in the clinical study and development of novel medicines for DM treatment from these frequently evaluated MPs.

## 1. Introduction

Diabetes mellitus (DM) is a metabolic disorder characterized by a persistent rise in blood glucose level (BGL) caused by ineffective insulin function, secretion, or both on target tissues [1–3]. Chronic hyperglycemia is associated with lifelong microvascular (retinopathy, neuropathy, and nephropathy) and macrovascular (coronary and peripheral arterial diseases and stroke) complexities, which are the typical features in all forms of DM. These complications result in damage and death of various organs that are diagnosed very late or have inappropriate medical follow-up [4–6].

Currently, it becomes one of the leading public health problems and the cause of morbidity and death universally [1, 2, 7–9]. In 2019, the International Diabetes Federation (IDF) estimates that 463 million (9.3%) adults aged 20–79 worldwide are currently living with diabetes. The total number is predicted to rise to 578.4 million (10.2%) by 2030 and 700.2 million (10.9%) by 2045 [10] with the highest increment in regions where economies are moving from low- to middle-income status without sufficient action to address the pandemic [1, 7, 10–12]. The number of deaths resulting from diabetes and its complications in 2019 is estimated to be 4.2 million [10].

Nearly 79.4% of diabetic patients live in low- and middle-income countries [10]. Around 19.4 million aged 20–79 had DM in Africa region with a prevalence of 3.9% in 2019. The Africa region is estimated to have the highest future increase in the number of people with diabetes compared to other parts of the world. By 2030 and 2045, there will be 28.6 million (47.5% increase), and 47.1 million (142.9% increase) adults aged 20–79 with diabetes, respectively, more than double the number in 2019 and the highest increase compared to other IDF regions [10].

Among the highly populated African nations that have the highest number of people with diabetes, including South Africa (4.6 million), Nigeria (2.7 million), the Democratic Republic of Congo (1.8 million), and Ethiopia (1.7 million). More than half (55.8%) of all 20–79-year-old adults with diabetes in the region live in one of these four countries [10]. Studies in various parts of Ethiopia showed that the prevalence of diabetes varies from 0.3 to 7.0% [13]. The IDF estimated that the total health expenditure due to diabetes in 2019 was USD 760 billion worldwide. It is projected that expenditure will reach USD 825 billion by 2030 and USD 845 billion by 2045 [10].

### 1.1. Challenges in the Treatment of DM

The existing approaches to the management of DM relied on keeping BGLs with normal limits via administration of appropriate medications together with lifestyle modifications [2, 14]. So far, the accessible medicines for DM are various preparations of insulin and oral antihyperglycemic agents [15–19]. The older oral hypoglycemics are sulphonylureas, alpha-glucosidase inhibitors, thiazolidinediones, and biguanides [19, 20] while the newer medicines include incretin-based therapies, sodium-glucose cotransporter 2 (SGLT-2) inhibitors, glucokinase activators, and injectable glucagon-like peptide (GLP-1) agonists [21]. These medicines are used either as monotherapy or in combination to achieve better treatment outcomes [19].

The conventional and newer agents are still with their shortcomings, and successful treatment of diabetes is being a global challenge requiring further investigations. In fact, these medications are associated with unnecessary drug reactions or side effects [3, 19] including hepatocellular injury, exacerbate renal diseases, blood dyscrasias, gastrointestinal irregularities, hypoglycemias, hypersensitivity reactions, weight gains, and lactic acidosis [22], which decrease their effectiveness and compliance rates [4, 22]. For instance, 3.9 and 32.7% severe and nonsevere hypoglycemic events were reported in 826 (2.8%) patients during their most recent year of sulfonylurea treatment, respectively [23]. Clearly, the weight gain associated with the use of thiazolidinediones (pioglitazone and rosiglitazone), sulphonylureas (glibenclamide), and insulin is also the major drawback for treating diabetes [24].

Several drawbacks have been reported associated with the use of antihyperglycemic medications, such as decreasing effectiveness and increasing adverse effects and toxicities. For instance, sulphonylureas lose their efficacy after six years of therapy in nearly 44% of patients, while BG lowering medicines are stated to be unable to control hyperlipoproteinemia [25]. Due to numerous limitations related to the use of existing synthetic BG-lowering medicines, the search for newer antihyperglycemic medicines from natural sources continues [25].

In spite of the extensive improvement made in the management of DM using various antihyperglycemic medicines in the past, the results of treatment are still far from successful. Because of the limitations of these agents, there remains a clear need for the identification of new antidiabetic drugs. Therefore, seeking extra safe and effective antidiabetic medicines from plant sources is becoming an active area of research in the scientific community. There are limited studies in Ethiopia, which compile *in vivo* and *in vitro* evaluations of extracts from medicinal plants (MPs) for antidiabetic potentials as a guide for future clinical trials and other related investigations. Therefore, this review provides a summary of MP extracts used for the treatment of DM.

### 1.2. Medicinal Plants (MPs) as Potential Sources of Antidiabetic Drugs

Internationally, MPs have been used as sources of medicines, and more than 80% of people depend on them using their extracts for their primary health care desires [26, 27]. Plant-based formulations become the key players of all available treatments due to accessibility, affordability, and minimum adverse effects, particularly in rural parts [19, 25].

Globally, WHO has estimated that more than 1,200 MP species are used in treating DM, mainly in developing nations [28], which represent above 725 genera in 183 families. Although, nearly 350 of them have been identified to possess antihyperglycemic effects, and few of them still require extensive investigations to prove their safety and effectiveness in humans [28].

In developing nations, especially MPs are used in treating DM to overcome the economic burden of medicines to the people [25, 29–31]. These days, treating DM using MPs is recommended [32] since these MPs contain various phytoconstituents such as flavonoids, terpenoids, saponins, carotenoids, alkaloids, and glycosides, which might have effects [25]. The combined effects of biologically active constituents (such as polyphenols, carotenoids, and glucosinolates) also result in the potential valuable properties of each MP, and this can act as the first stage to understand their biological effects and beneficial activities [25, 33, 34].

Numerous MPs are well documented for their medicinal values to treat DM in the traditional methods of medications. Though some of them have been studied scientifically for their hypoglycemic effectiveness [29, 35], many active constituents were isolated from MPs for direct uses as medicines or represent as lead compounds or pharmacological drugs. For instance, metformin, a BG-lowering medicine, was isolated from MP *Galega officinalis* L., which was used historically in primitive Europe in treating DM [19, 36].

Many studies revealed the uses of MPs with antihyperglycemic activities in the treatment of DM [14]. The activities of these MPs could delay the progress of DM complications and ameliorate the metabolic defects. During the past few years, some of the new bioactive medicines isolated from antihyperglycemic MPs indicated BG-lowering effects with better efficiency than oral antihyperglycemic medicines used in clinical treatment [14].

### 1.3. Possible Mechanism of Actions of Medicinal Plants in Treating DM

Bioactive compounds that are obtained from various MPs have been reported to have potent BG-lowering potentials [37–45]. The mechanism of lowering BGL might be a result of activation of releasing insulin from *ß*-cells, reduction of glucose absorption, stimulation of glycogenesis, and/or enhancement of glucose use [28, 46, 47]. In addition to lowering BGL, secondary metabolites obtained from MPs have the capacity to restore the impaired *ß*-cells and terminate oxidative stress on *ß*-cells [35, 37–40].

Furthermore, inhibiting cellular apoptosis, reducing renal glucose reabsorption [37, 38, 45], enhancing the metabolic rate of oxygen consumption [45], and promoting glucose transporter (GLUT-2) expression and translocation of GLUT-4 [38, 43] are also important mechanisms illustrated with certain secondary metabolites that are responsible for antidiabetic effects [38, 43]. Blocking pancreatic *β*-cell K^+^ channel [48], stimulating cyclic adenosine monophosphate (cAMP), and providing some essential elements (calcium, zinc, magnesium, manganese, and copper) for the *β*-cell [37] are also some mechanisms that are possibly participated in *β*-cell dysfunction found in DM [37, 48].

Blocking the actions of *α*-amylase and *α*-glycosidase enzymes, which are essential for carbohydrate digestion, is used as an optional treatment approach for type 2 diabetes. Folkloric MPs with antihyperglycemic effects via inhibition of these enzymes and their free radical scavenging potentials are becoming promising modalities in treating type 2 DM and associated complications [41]. Medicinal plants have central roles to discover newer medicines and have begun to get greater attention as sources of bioactive constituents as well as antioxidants. Antioxidant activity has a protective effect in restoring *β*-cell function in diabetes. Because free radicals are known to damage and mutation of cells, and hence, oxidative stress has a vital role in the pathogenesis of DM and its complications, MPs with antioxidant effect will have paramount importance in treating diabetes and its complications via scavenging free radicals [37].

### 1.4. Important Phytomolecules for Treatment of DM

Phytomolecules that are obtained from various MP sources containing flavonoids, phenolic compounds, alkaloids, terpenoids, saponins, tannins, glycosides, glycolipids, dietary fibers, carotenoids, and anthocyanins have demonstrated potential BG-lowering activities [38] through different mechanisms [43].

Flavonoids and other polyphenols show BG-lowering activities by enhancing GLUT-2 expression in pancreatic *β*-cells [38, 43, 49], enhancing insulin release [25, 46, 49–51], and increasing expression and promoting translocation of GLUT-4 [25, 38, 43, 49, 51], which can increase glucose uptake by the muscle, liver, and adipose tissue [49, 50]. Flavonoids also regenerate pancreatic beta cells [37, 43, 47, 52–54], reduce aldose reductase [46], increase calcium ion uptake [46], retard the gastric emptying rate [46], and inhibit *α*-glycosidase [46, 49, 54] and *α*-amylase [43, 49]. In addition, they have antiapoptotic activities [25, 49].

Tannins [35, 53] and phenols might contribute to BG-lowering activities due to their potential to stimulate insulin secretion or possess insulin-like effects [43], reduce carbohydrate absorption by impeding *α*-glucosidase and *α*-amylase [35, 43], enhance *β*-cells propagation and restoration [43], and prevent *β*-cells impairment through free radical scavenging effects [43, 55, 56].

Saponins exhibit their antihyperglycemic effects through the probable mechanisms of protecting pancreas *β*-cells, stimulating insulin release/secretion [35, 46, 57, 58], and ameliorating insulin resistance [58].

Triterpenoids seem to have promising antidiabetic properties that can be achieved through the inhibition of *α*-glucosidase, *α*-amylase [59, 60], aldose reductase, hepatic glycogen phosphorylase, and sweetness [60]. They have also agonistic properties of emerging G-protein-coupled receptor (TGR5). Additionally, they prevent pancreatic *β*-cell dysfunction, increase insulin-stimulated GLUT-4 translocation, and decrease oxidative stress and body weight. Some triterpene compounds possess the ability to suppress the formation of advanced glycation end products (AGEs) and are promising agents in the prevention and treatment of DM complications [60].

Alkaloids, in recent years, have received extra attention due to their potential role in the treatment of diabetes through inhibition of *α*-glucosidase, *α*-amylase [59, 61], dipeptidyl peptidase-4 (DPP-4), and AGEs and by possessing potent protein tyrosine phosphatase 1B (PTP1B) inhibitory effects [61]. They activate 50 adenosine monophosphate-activated protein kinases (AMPK) and GLUT = 4 translocation. Alkaloids are effective for pancreatic regeneration and insulin release. They also show protective effects on oxidative tissue damage [61, 62].

Potential BG-lowering substances from plant extracts, which are used for many diseases with identified drug targets and proven safety, are required in treating diabetes [38, 62–64]. Therefore, developing new antihyperglycemic agents from plant-derived substances, which are easily available, seems highly attractive research areas as currently accessible medicines have drawbacks regarding safety, efficiency, and affordability [65, 66]. Effective novel compounds with multiple targets, BG-lowering effect, and proven continuous safety have to be targeted in clinical settings for users with concomitant pertinent lipid and glucose metabolic abnormalities. Consequently, this paper opens the way to develop medicines in treating chronic multigenic metabolic and cardiovascular disorders, for which therapy is currently inadequate or nonexistent [63, 67, 68].

Therefore, the attempt of our work is to look at different *in vitro* and *in vivo* antidiabetic evaluations of MPs in Ethiopia. Furthermore, this work could pave a way for other complementary studies plus the development of numerous accessible and inexpensive antidiabetic phytomedicines and provide direction for future clinical and other related investigations.

## 2. Methods

We have searched relevant articles to the scope of our work using Google Scholar, Medline, Web of Science, SCOPUS, and PubMed databases to extract *in vitro* and *in vivo* BG-lowering agents' investigations of MPs conducted in Ethiopia. The search terms used were “antidiabetic activity,” “antidiabetic effect,” “antidiabetic potential,” “antihyperglycemic activity,” “antihyperglycemic effect,” “medicinal plants,” “hypoglycemic effect,” “hypoglycemic activity,” “blood glucose-lowering effect,” and “diabetes in Ethiopia.” Only studies conducted in Ethiopia were included using English keywords. If studies were done in Ethiopia plus other countries, the data obtained from Ethiopia were used. Original articles and studies written in English were included, whereas studies whose full articles were not accessible online were excluded from the study. We thereby provide scientific evidences based on evaluations on MPs as potential sources of antidiabetic drugs in Ethiopia.

**Figure 1:**
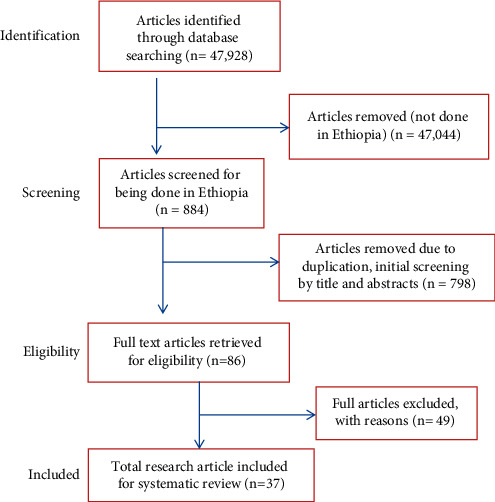
PRISMA flow diagram showing the selection process.

The identification of records, screening of titles and abstracts, and evaluation of eligibility of full texts for final inclusion was conducted in accordance with the Preferred Reporting Items for Systematic Review and Meta-Analysis (PRISMA) flow diagram [69]. Research papers available online before November 30, 2020, were considered for our study. A total of 47,928 articles were identified through database searching. All articles not done in Ethiopia were removed, and we obtained 884 articles. Eighty-six (86) research articles were obtained after removing the 798 duplicate articles, following the initial screening by titles and abstracts. Finally, as shown in Figure 1, these full-text articles were assessed for eligibility, and data were extracted from the remaining 37 experimental investigations of MPs. All research articles were summarized in the table and figures clearly with the key information and findings.

## 3. Results

### 3.1. Pharmacological Investigations

Overall, 37 MP species belonging to 19 families were investigated that showed potential antidiabetic activities *in vitro* or *in vivo* in Ethiopia as presented in Table 1.

From these families, Lamiaceae accounted the greatest number of species followed by Aloaceae and Fabaceae. Medicinal plants belonging to the Lamiaceae family have many active phytochemical substances [11, 46, 47, 54, 77–80]].

**Figure 2:**
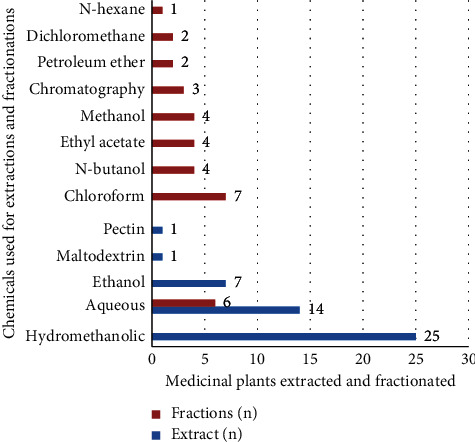
Extracted and fractionated medicinal plants used for antidiabetic activities in Ethiopia.

The antidiabetic effects of crude extracts and fractions of different MPs parts using different chemicals were conducted as presented in Figure 2. Most experiments were performed with hydromethanolic extracts to get a greater percentage of extract yield based on previous studies conducted. Importantly, 80% methanol is more efficient in the cell wall and seed degradation as well as having low or no enzyme activity as compared to water. Additionally, the methanol extract of plant materials contains a wide variety of polar (and moderately nonpolar) compounds [70, 91, 92].

**Figure 3:**
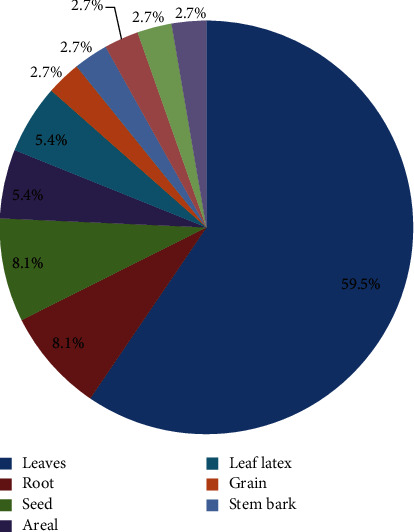
Medicinal plants parts experimentally used for antidiabetic activities in Ethiopia.

Among chemicals used for crude extraction of aqueous, methanol was mostly used, followed by the use of aqueous. Mostly, solvent fractionations were carried out using chloroform. Different MPs parts were used experimentally for antidiabetic activities in Ethiopia. Among these, leaves were the most commonly experimentally evaluated plant parts followed by roots and seeds as shown in Figure 3.

Pharmacological studies on antidiabetic activities of MPs, which have been investigated directly or indirectly in Ethiopia, are presented in Table 1. Among MP species studied experimentally, *Thymus schimperi* Ronniger (Lamiaceae), *Moringa stenopetala* Baker f. (Moringaceae), *Ajuga remota* Benth (Lamiaceae), and *D. stramonium* Linn. (Solanaceae) were the most commonly used MPs in treating DM as discussed below.

## 4. Discussion

Numerous MPs are being used traditionally for the management of DM in Ethiopia for a long period though the number of plants studied is limited. This review summarized studies conducted and emphasizing the need for further investigations. Potential plant-derived antidiabetic compounds that can act on multiple disease-related drug targets with proven long-term safety are needed for the treatment of DM. Natural products are promising lead candidates for discovering and also easily available, affordable, and tolerable [19, 25, 38]. Bioactive compounds that are obtained from various medicinal plant sources have been reported to have potent antidiabetic activity [38, 43, 46, 55–62, 67, 79, 82, 85, 86] through different mechanisms as we have discussed below in the text. The effect could act either individually and/or synergistically.

### 4.1. *Thymus schimperi (T. schimperi)* Ronniger (Lamiaceae)

T. schimperi, locally known as Tosign in Amharic, is endemic to Ethiopia [46]. The antidiabetic activity of methanol extract plus butanol and the aqueous fraction of *T. schimperi* leaves were tested in both normal and STZ-induced diabetic mice. Significant reduction of BGL at all given doses was seen with methanolic crude extract in diabetic mice and was in a dose-dependent way (250 mg/kg (14.76 ± 6.1%), 500 mg/kg (25.12 ± 11.5%), and 750 mg/kg (27.15 ± 10%)). Percentage reduction of BGL was shown in *n*-butanol fraction at 500 mg/kg (36 ± 7.3%) and 250 mg/kg (22.2 ± 4.3%) doses. The aqueous fraction with the dose of 250 and 500 mg/kg also decreased the BGL by 17.6% ± 6.0% and 18.4 ± 5.0%, respectively [46].

Aqueous and 80% methanolic extracts of *T. schimperi* Ronniger leaves with the same dose 500 mg/kg indicated substantial reduction of BGL (*P* < 0.05) at 14 days in alloxan-induced diabetic mice. Significant reduction of BGL in 80% methanol 500 mg/kg (*P* < 0.001), aqueous 250 mg/kg, and 80% methanol extracts (*P* < 0.05) was also detected at 21 days [2]. Other investigations, *α*-amylase and glucosidase inhibitory assays, were conducted in 80% methanol and boiling water leaves extracts of *T. schimperi*. The *α*-amylase inhibitory activity at 2.5 mg/mL of the 80% methanol and boiling water extracts were 68.6 ± 5.9% (IC_50_ 0.33 ± 0.05 mg/ml) and 48.7 ± 7.1% (IC_50_ 2.24 ± 0.53 mg/ml) at *P* < 0.05, respectively. At the dose of 2.5 mg/mL, both hot water and 80% methanol extracts also revealed *α*-glucosidase inhibition activities 96.8 ± 10.5% (IC_50_ 0.69 ± 0.04 mg/ml) and 84.4 ± 8.5% (IC_50_ 0.05 ± 0.01 mg/ml) at *P* < 0.05, respectively [79]. Bioactive constituents that are obtained from T. *schimperi*, such as alkaloids, polyphenols [46], flavonoids [46, 79], tannins, saponins [46], terpenoids [2, 46], and phenols [79], have been reported to have potent BG-lowering potentials.

The results of the studies indicated the potential antihyperglycemic effect of the extract due to many explanations with these findings. The inhibition of *α*-amylase activity describes one of the treatment methods commonly used for the prevention and control of hyperglycemia in type 2 DM patients after food intake by dropping the absorption of glucose [2], which involved the delaying of postprandial hyperglycemia [2, 79]. This result agreed with other *in vitro* evaluations of the plants [35, 37, 41, 90]. The antidiabetic result of thymus plants is also due to *α*-glucosidase enzyme inhibitory effect and antioxidant activity *in vitro* of the plant [2, 79] in line with *in vitro* study of *T. vulgaris*^79^. These *α*-amylase and *α*-glucosidase activities may be due to the presence of alkaloids [59, 61], triterpenoids [59, 60], tannins [35, 43], and flavonoids [49]. Furthermore, *T. schimperi* may prevent the destruction or regenerate the destroyed beta cells of the pancreas by the presence of different secondary constituents, triterpenoids [60], saponins [35, 46, 57, 58], tannins [43, 55, 56], flavonoids [38, 43, 49], and polyphenolic [2] and contribute to a significant BGL effect. Thus, the antidiabetic result of the *T. schimperi* extract may be associated with the existence of different secondary metabolites in plants. Therefore, these properties might be contributed to the clinical study of *T. schimperi* leaves extract.

### 4.2. *Moringa stenopetala (M. stenopetala)* Baker f. (Moringaceae)

*M. stenopetala*, Shiferaw in Amharic, which is endemic to East African countries mainly located in southern parts of Ethiopia [67, 82] and northern Kenya [82]. The effects of aqueous extract and an isolated fraction of *M. stenopetala* leaves in normal and alloxan-induced mice models were examined by Mussa et al. [82]. Crude aqueous extract (except at 6 h), chloroform, *n*-butanol, and aqueous residue fractions significantly (*p* < 0.05) reduced BGLs at all periods. *N*-butanol and aqueous residue fractions significantly (*P* < 0.005) decreased BGLs at 1.5 h of their administrations.

Ethanol and aqueous crude extracts, petroleum ether, chloroform, and butanol fractions of the leaf extracts of M. stenopetala were also tested in normoglycemic and aloxan-induced mice models. The ethanol extract significantly lowered BGL at 60, 180, and 240 mins (*P* < 0.01) and 120 mins (*P* < 0.001) of single dose in normoglycemic mice. Aqueous and chloroform extracts showed the same reduction of BGLs at 120 mins (*P* < 0.01), 180 mins (*P* < 0.05), and 240 mins (*P* < 0.05). Likewise, butanol fraction significantly (*P* < 0.05) decreased starting from 180 mins. The ethanol extracts significantly reduced BGL at 60 mins (*P* < 0.05) as well as 120, 180, and 240 mins (*P* < 0.001). Aqueous extract also reduced BGL significantly (*P* < 0.01) at 120, 180, and 240 mins (*P* < 0.001) of single dose in alloxan-induced diabetic mice. After administration of ethanol and aqueous extracts, significant reduction was resulted in BGL starting from the 3rd (*P* < 0.001) and on the 5th day (*P* < 0.01). Chloroform and butanol fractions showed meaningful changes on the 5^th^ day (*P* < 0.01) and 8th day (*P* < 0.001) of repeated doses in alloxan-induced mice model [83].

According to another study, aqueous ethanol and *n*-butanol extracts of *M. stenopetala* leaves 500 mg/kg significantly decreased BGL (*P* < 0.05) in STZ-induced rat model after 14 days. The extracts also decreased postprandial BGL (*P* < 0.001) at 750 mg/kg dose [67]. The maltodextrin (9%) and pectin (1%) of leaves extract of *M. stenopetala* had also shown significant (*P* < 0.05) decrease in BGL with dose-dependent manner in the same model [84].

The antihyperglycemic activity of *M. stenopetala* was supported by leaves extraction and fractionation in alloxan-induced male diabetic mice. Significant BGL reduction (*P* < 0.01) was seen with hexane fraction at 2 and 4 h whereas dichloromethane fractionate was seen at 2, 4, and 6 h of administrations, respectively. Decreasing BGL with butanol fraction was exceedingly significant *P* < 0.001 at 2 and 4 h, while aqueous residue was very significant *P* < 0.01 at 2 h and *P* < 0.05 at 4 h of administrations. Chromatographic fraction 2 and 3 showed very significant BGL reduction (*P* < 0.01) at 3 h after administrations. Chromatographic fraction 1 reduced BGL significantly (*P* < 0.05) at 6 h after administration [85].

Another investigation was also done to describe the antiglycation effect of hydroalcoholic leaves extract in bovine serum albumin (BSA)/fructose method. The *M. stenopetala* leaves extract significantly (*P* < 0.05) inhibited advanced glycation end products (AGEs) formation by 54.75 ± 0.94% at 2 mg/ml. Besides, the extract decreased the concentration of fructosamine, formation of N*ε*-(carboxymethyl) lysine (CML), and the extent of amyloid cross *β*-structure in fructose-induced BSA glycation test [86].

Alkaloids, saponins, glycoproteins, amino acids and proteins [82], polyphenols [67, 85, 86], flavonoids [67, 85], phenolic compounds, and glycosides [85] were the major findings that could also have antidiabetic potentials through different mechanisms as cited above. The previous study on the chemical composition of the leaves of *M. stenopetala* revealed the presence of rutin, 4-(4′-0-acetyl-*α*-L-rhamnosyloxy)-benzyl isothiocynate, 4-(4′-0-acetyl-*α*-L-rhamnosyloxy)benzaldehyde, and O-(rhamnopyranosyloxy)-benzyl glucosinate [82, 85, 93].

The results of the findings indicate that the extracts of *M. stenopetala* produced potential antihyperglycemic effects due to various descriptions. It produced regeneration/proliferation of the pancreatic *β*-cells possibly due to the prevention of free radical formation [67, 84]. Antiglycation activities may be associated with the presence of a high amount of phytochemicals such as polyphenolic compounds in the plant materials. Aggregation of advanced glycation end products may result in pancreatic islet amyloidosis that causes the damage of *β*-cell and compromised insulin secretion. Hence, the extract suppressed the formation of amyloid cross-*β* structure of fructose-induced BSA. The antioxidant activity of the extract also contributes to antiglycation activity [86]. These beneficial effects of the extract of *M. stenopetala* may ameliorate risks of degenerative diseases in diabetic patients. Therefore, due to these investigations, the extracts of *M. stenopetala* could be an attractive source of alternative treatment and for further clinical studies.

### 4.3. *Ajuga remota* Benth (*A. remota*) (Lamiaceae)

*A. remota* grows widely in East Africa (different regions of Ethiopia) [78], Saudi Arabia, Yemen, and Afghanistan to East Asia [11, 77]. Different investigations validated the antidiabetic activity of *A. remota* Benth in alloxan diabetic mice [11, 77] and STZ-induced diabetic rats [78]. The fasting mean BGL of alloxan-induced mice model treated with aqueous leaves extract 300 and 500 mg/kg and ethanol extract 300 and 500 mg/kg was reduced by 27.96%, 38.98%, 28.09%, and 28.25%, respectively (*P* < 0.05) [11].

Another investigation showed the hypoglycemic effect of this plant in alloxan-induced mice model. Aqueous extract of leaves 300 and 500 mg/kg decreased BGL by 27.83 ± 2.96% and 38.98 ± 0.67% (*P* < 0.0001), respectively. Seventy percent ethanol extract 300 and 500 mg/kg also caused a reduction of 27.94 ± 1.92% and 28.26 ± 1.82% (*P* < 0.05), respectively [77].

Assefa et al. [78] showed the ethanolic leaves extract of *A. remota* can have a hypoglycemic effect in the STZ-induced rat model. The dose of 200 and 400 mg/kg ethanol leaves extract significantly decreased (*P* < 0.05) fasting BGL on the 21st and 14th day of treatment, respectively. Steroids, phenols, flavonoids [11, 77, 78], tannins [11, 77], saponins, [77, 78], diterpenoids, phytoecdysteroids, and glycosides [78] were major secondary metabolites that could support the antidiabetic activities of this MP.

A significant number of compounds have been isolated from various species of the *Ajuga* herb including sterols (ajugalactone, *β*-sitosterol, *γ*-sitosterol, and stigmasterol), phenolic components, arabinose, cerotic acid, ecdysterone, phytoecdysteroids (phytoecdysteroid, cyasterone, ajugalactone, and ajugasterone A–C), flavonol glycosides, triterpenoid (ergosterol-5,8-endoperoxide), iridoid glycoside (8-O-acetylharpagide, 6,8-diacetylharpagide, ajureptoside, 8-acetylharpagide, and harpagide), neoclerodane-diterpenes, and diterpenes (ajugarins I, II, IV, and V) [11, 94–97]. Epicatechin (flavonoids), catechin (tannin), and vindoline (an alkaloid) were some of the documented compounds that were isolated from the plant with the potential to decrease the blood glucose level [11, 77].

The mechanism of antidiabetic effects of the extracts of *A. remota* leaves might be due to the presence of well-known antioxidant phytochemicals such as flavonoids, polyphenols, tannins [11, 43, 55, 56], and triterpenoids [60–62], which acts as free radical scavengers. The presumed mechanism of action of these antioxidants was because of an insulin-mimetic effect on the peripheral tissues by either stimulation of regeneration process or release of pancreatic secretion of insulin from existing *β*-cells [11, 78]. The activities also might be due to inhibition activity against *α*-glucosidase enzymes in the small intestine [78]. Thus, the significant antidiabetic effect of the extracts of *A. remota* could be due to the presence of these properties in the extracts, which could act synergistically and/or independently to enhance the activity of glycolytic enzymes.

### 4.4. *Datura stramonium (D. stramonium)* Linn. (Solanaceae)

*D. stramonium* is widely distributed throughout the world, including Ethiopia [43]. Belayneh et al. [38] and Melaku and Getnet [43] stated the hypoglycemic activity of hydromethanolic root and seed extracts of *D. stramonium*, respectively, in animal models. The hydromethanolic root extracts of *D. stramonium* at doses 100 and 200 400 mg/kg (*P* < 0.05) and 400 mg/kg (*P* < 0.01) meaningfully decreased BGL at 1 and 2 h postprandial glucose test. The 100 and 200 400 mg/kg (*P* < 0.05 on day 7 and 14, respectively) and 400 mg/kg (*P* < 0.05 on day 7 and *P* < 0.01 on day 14) significantly decreased BGL in STZ-induced mice model [38].

The administration of hydromethanolic seed extract from *D. stramonium* significantly reduced BGL (*P* < 0.05 at dose of 100 mg/kg and *P* < 0.01 at doses of 200 and 400 mg/kg) in STZ-induced diabetic mice. At all strengths of seed extract significantly (*P* < 0.0l) decreased BGL on 7th and 14th day. Seed extract at doses of 200 and 400 mg/kg also meaningfully (*P* < 0.05) ameliorated body weight of diabetic mice on these days [43].

Flavonoids, phenols, tannins, steroids, glycosides, alkaloids [38, 43], anthraquinones [38], saponins, and terpenoids [43] are also reported which could have antidiabetic activities.

Hydromethanolic root extract showed significant antihyperglycemic activities possibly due to pancreatic *β*-cell regeneration or protective effects due to their antioxidant activities [38]. This was revealed by different studies of plant extracts [11, 35, 78, 98]. Leaf extract of *in vitro* study of this plant supported this activity [99] Crude aqueous leaf extract and methanolic seed extract of *D. stramonium* possess *in vitro α*-amylase enzyme inhibitory activity suggesting the plant to be a potential candidate for DM [43, 100, 101]. Significant improvement in body weight by the plant extract might have protective effects against protein catabolism and muscle wasting possibly due to the enhancement of insulin secretion and/or action [38]. Additionally, the plant extract may have a direct effect on lipid absorption and metabolism that can lead to the improvement of diabetic dyslipidemia [38, 102]; hence, augmenting insulin secretion and/or action is a potential approach to treat this disorder [38]. Therefore, extracts from plant material of *D. stramonium* have the potential antidiabetic activities, which can be the indication for further clinical investigation.

### 4.5. Clinical Studies of *Trigonella foenum-graecum* L. (Fenugreek)

*Trigonella foenum-graecum* is a useful MP belonging to the family Fabaceae distributed throughout the world including Ethiopia [103]. *T. foenum-graecum* seed powder solution (by the administration of 25 mg for 30 consecutive days) significantly improved dyslipidemia in newly diagnosed type II diabetic patients as dyslipidemia is common in type 2 diabetes, as both insulin deficiency and resistance affect enzymes and pathways of lipid metabolism [103]. Disturbances of lipid metabolism appear to be an early event in the development of type 2 diabetes [104]. It showed significantly (*P* < 0.001) lower total cholesterol level by 13.6% as compared with the baseline level and the control group. Triglyceride level also reduced by 23.53% compared with the baseline level (*P* < 0.001) and compared with the control group (*P* < 0.05). Low-density lipoprotein cholesterol level also reduced by 23.4% as compared to the baseline level (*P* < 0.001) and the control group (between groups) (*P* < 0.001), but the treatment group showed significantly increased high-density lipoprotein cholesterol level by 21.7% as compared to the baseline level, within group (*P* < 0 001), and the control group, between groups (*P* < 0.001). Therefore, the seed powder solution showed a significant reduction in total cholesterol, low-density lipoprotein cholesterol, and triglycerides levels and an increase in high-density lipoprotein cholesterol level. Hence, it had pronounced effects in improving lipid metabolism in type II diabetic patients with no adverse effects [103]. Different clinical trials have shown that plants cause significant antidiabetic and antidyslipidemic activities in humans [105–108]. Therefore, *T. foenum-graecum* seed may provide new alternatives for the clinical management of type II diabetes.

## 5. Limitations of the Study

Even if we used all previous findings that were done in Ethiopia, there are some limitations that might limit the results of this work. The methodological quality (alloxan or STZ induction method induces mostly type 1 DM), which Methodological quality could be a challenge since there might exist an overlap between type 1 and type 2 DM during diabetic induction with aloxan or STZ. Use of terminology when searching evidence might be the other limitation.

## 6. Conclusion and Perspectives

Medicinal plants have provided clinically beneficial sources of antidiabetic medicines. Due to the rising of multidrug-resistant DM, adverse drug reactions, and toxicities of currently available conventional drugs, TMs might be indispensable, inexpensive, and alternative potential sources of therapy. Therefore, an urgent need for the development of novel medicines in treating DM is required. Using confirmed investigation systems, the potential of MPs to produce new antidiabetic medicines has been supported with *in vitro* and *in vivo* studies in Ethiopia, as shown in this paper. Biological investigation of MPs used traditionally in primary health care is among evidences in which searching for new leading compounds should focus on. Preliminary phytochemical constituents and toxicity profiles of MPs have also been evaluated to some extent to assure their safety profiles even though they need further investigations. Hence, it is crucial to identify their active constituents and validate the efficacy and safety of MPs. Studying the effects of unexplored MP species on DM, isolating the active substances, evaluating the possible mechanisms of actions of extracts to obtain further data on the pharmacological and clinical effects, and exploring additional new lead compounds and drugs for the treatment of DM are also needed in more detail.

Consequently, this paper provides baseline data to other researchers of MPs that have the potential BGL-lowering effects and scientific knowledge in Ethiopia. Further investigations for antidiabetic medicines discovery and development should also focus on pharmacokinetic profiles of the promising candidates studied so far in the area.

Towards the assessments of other beneficial properties of these MPs in DM treatment should be directed. The development of more effective, reasonably priced, and standardized phytopharmaceuticals in close cooperation with clinicians and industries would also be advisable. Therefore, extracts from the leaves of *T. schimperi* Ronniger (Lamiaceae), *M. stenopetala* Baker f. (Moringaceae), and *A. remota* Benth (Lamiaceae) and root and seed of *D. stramonium* Linn. (Solanaceae), which were mostly investigated in treating DM in Ethiopia may comprise interesting samples for development of new drugs. Besides, more pharmacological (pharmacodynamic and pharmacokinetic parameters) and toxicological investigations should be pursued to validate the safety of these MPs' source constituents. Finally, we recommend upcoming researches to ensure future success in the clinical studies and development of novel medicines for DM treatment from these commonly evaluated MPs.

## Figures and Tables

**Table 1:** Medicinal plant extracts evaluated *in vitro* and *in vivo* for antidiabetic activities in Ethiopia.

Family/species	Plant crude extracts and fractions used	Models used		Dose(s) (mg/kg) BW	Standard drug used	Phytochemical constituents	Effects	LD_50_ (mg/kg)	Ref
		*In vitro*	*In vivo*						
Acanthaceae (1) *Acanthus polystachyus* Delile	80% methanol root extract		STZ-induced diabetic rats	100, 200, and 400	GL	Tannins, flavonoids, saponins, polyphenols, terpenoids, glycosides, and anthraquinone	↓BGL (*P* < 0.05) at the extract (100, 200, and 400 mg/kg) for 28 days.	>2,000	[70]
(2) *Justicia Schimperiana* T.Anderson	Aqueous leaf extract		Normal and STZ-induced diabetic mice	200 and 400	GL	Alkaloids, phenols, and terpenoids	Showed significant tolerance (*P* < 0.05) at 1 and 2 h. ↓BGL (*P* < 0.05) at 4 h in normoglycemic mice. ↓BGL (*P* < 0.05) at 400 mg/kg extract at 2, 3, and 4 h of treatment in diabetic mice.	>2,000	[71]
Aloaceae (3) *Aloe debrana* Christian	Ethanolic extract of leaves		STZ-induced diabetic rat	300	GL	ND	Showed antidiabetic activity (*P* < 0.05).	ND	[72]
(4) *Aloe megalacantha* Baker	Leaf latex extract		STZ-induced diabetic mice	100, 200, and 400	GL	Alkaloids, flavonoids, phenols, tannins, saponins, glycosides, anthraquinone, and terpenoids	↓BGL (*P* < 0.05 and *P* < 0.001) with 100, 200, and 400 mg/kg doses at the 7th and 14th days, respectively.	>2,000	[18]
(5) *Aloe megalacantha* Baker	80% methanol and TLC fraction of leaves latex	Chromogenic DNSA		20, 40, 60, 80, and 100 *μ*g/mL	Acarbose	ND	Possessed *α*-amylase suppression activity at both the leaf latex and the fraction (*R*_f_ value of 0.49) with IC_50_ value of 74.76 ± 1.98 and 96.75 ± 1.98 *μ*g/mL, respectively (*P* < 0.001).	ND	[41]
(6) *Aloe monticola* Reynolds	80% methanol and TLC fraction of the leaves	Chromogenic DNSA		20, 40, 60, 80, and 100 *μ*g/mL	Acarbose	ND	Possessed *α*-amylase suppression activity at both the leaf latex and the fraction (*R*_f_ value of 0.27 and 0.61) with IC_50_ value 78.10 ± 1.88, 56.95 ± 1.88, and 64.03 ± 3.60 *μ*g/mL, respectively (*P* < 0.001)	ND	[41]
(7) *Aloe pulcherrima* Gilbert and Sebsebe	Leaf latex		Normoglycemic, glucose-loaded, and STZ-induced diabetic mice	200, 400, and 600	GL	Flavonoids, anthraquinone, saponins, glycosides, tannins, phenols, and alkaloids	Inhibited sucrase (IC_50_ = 2.92 *μ*g/ml), maltase (IC_50_ = 11.81 *μ*g/ml), and *α*-amylase (IC_50_ = 14.92 *μ*g/ml). ↓BGL (*P* < 0.05) in OGTT mice. ↓BGL of diabetic mice (*P* < 0.05) on week 1 and 2. ↓BGL with increasing the doses on week 1 (*P* < 0.05 (200 mg/kg), *P* < 0.01(400 mg/kg), and *P* < 0.001 (600 mg/kg)). Improved dyslipidemia and BW of diabetic mice (*P* < 0.05).	ND	[42]
Asteraceae (8) *Artemisia afra* Jacq. ex Willd	Aqueous and methanolic extract of aerial parts		Alloxan-induced diabetic Swiss albino mice	500, 750, and 1,000	GL	Tannins, saponins, chromophores, phosphosteroid, withanoids, flavonoids, and anthraquinone	↓BGL by 24% (*p* < 0.005) and 56.9% (*P* < 0.0004) at doses of 500 and 750 mg/Kg aqueous extract, respectively. ↓BGL by 49.8% (*P* < 0.0001) at dose of 1,000 mg/kg methanolic extract at 5 hr.	>5,000	[9]
(9) *Stevia rebaudiana* Bertoni	Ethanol and aqueous extract of leaf		Alloxan-induced diabetic mice	300 and 500	GL	Alkaloids, steroids, phenols, and flavonoids	↓BGL from 335.6 ± 14.01 to 234.00 ± 16.20 mg/dl at a dose of aqueous extract 300 mg/kg (*P* < 0.05). ↓BGL from 370.00 ± 19.46 to 221.2 ± 18.94 mg/dl at a dose of aqueous extract 500 mg/kg (*P* < 0.05). ↓BGL by 28.71 and 33.04% at doses of ethanol extract 300 and 500 mg/kg, respectively (*PP* < 0.05)	>5,000	[11]
Celastracea (10) *Catha edulis* Forsk	Fresh juice stem tips and leaves with water		STZ-induced diabetic rats	4.5 ml/kg	GL	ND	↓Fasting BGL from 223.7 ± 27.6 to 106 ± 18.2 mg/dl, at the end of study (*PP* < 0.05).	ND	[45]
Combretaceae	Methanol extract, chloroform, ethyl acetate, and *n*-butanol fraction of stem bark	Chromogenic DNSA		10, 50, and 100 *μ*g/mL	Acarbose	Tannins, saponins, polyphenols, flavonoids, terpenoids, and steroids	The crude extract and solvent fractions showed a dose-dependent *α*-amylase inhibitory activity. Highest *α*-amylase inhibitory potential with the lowest IC_50_ value of 63.41 *μ*g/mL by chloroform fraction.	>2,000	[1]
(11) *Terminalia brownii* Fresen.			Normoglycemic, OGTT, and STZ-induced diabetic mice	250, 500, and 750	GL		↓BGL by 20.8, 28.2, and 32.6% after 4 h of treatment of crude extract at dose of 250, 500, and 750 mg/kg, respectively, in normoglycemic mice. ↓Hyperglycemia with OGTT by the crude extract at a dose of 500 mg/kg (*P* < 0.01), 750 (*P* < 0.05) after 60 min, and 750 mg/kg (*P* < 0.01) after 120 mins. ↓BGL (*P* < 0.01) with ethyl acetate and aqueous fractions at 500 mg/kg in diabetic model.		
Euphorbiaceae (12) *Croton macrostachys* Hocsht. ex Del.	Hydroalcoholic root extract		OGTT and STZ-induced diabetic mice	100, 200, and 300	GL	Alkaloids, phenols, tannins, terpenoids, saponins, phlobatannins, and flavonoids	↓Hyperglycemia by 300 mg/kg compared to 100 (*P* < 0.001) and 200 mg/kg (*P* < 0.01) in diabetic mice. ↓BGL in OGTT at doses of 100 (*P* < 0.01), 200 (*P* < 0.001), and 300 mg/kg (*P* < 0.001) after 60, 90, and 120 mins of glucose loading.	>5,000	[73]
Fabaceae (13) *Calpurnia aurea* (Ait.) Benth.	80% methanolic seed extract		Normoglycemic, OGTT, and STZ-induced diabetic mice	2.75, 5.5, and 11	GL	Alkaloids, phenols, flavonoids, and terpenoids	↓BGL (*P* < 0.05) by 20.39% at 5.5 mg/kg at 6 h. ↓BGL by 32.72% and 46.11% with 11 mg/kg extract at 4 h (*P* < 0.01) and 6 h (*P* < 0.001) h, resPectively, in normoglycemic mice. ↓Hyperglycemia (*P* < 0.05) with 5.5 and 11 mg/kg at 2 hr in OGTT mice. ↓BGL with 2.75 (*P* < 0.05), 5.5 (*P* < 0.01) and 11 mg/kg (*P* < 0.001) extract on the 7th and 14th day of repeated doses in diabetic mice.	≥175	[52]
(14) *Calpurnia aurea* (Ait.) Benth.	Hydromethanolic leaf extract		STZ-induced diabetic mice	100, 200, and 400	GL	Phenols, alkaloids, terpenoids, and flavonoids	↓Hyperglycemia (*P* < 0.05) at all doses of extract (100, 200, and 400 mg/kg) at the 7th and 14th day of repeated daily dose administration.	>2,000	[66]
(15) *Lens culinaris*	80% methanol extract of seed		STZ-induced diabetic mice	100, 200, and 400	GL	Polyphenols, flavonoids, saponins, triterpenoids, phytates, lectins phytosterols, and defensins	↓BGL at all doses of the extract (*P* < 0.05) at days 7, 14, and 21. Antidiabetic activity (*P* < 0.05) exhibited by 400 mg/kg compared to 100 mg/kg.	>2,000	[74]
Medik (16) *Indigofera spicata* Forsk.	Hydroalcoholic crude extract of leaves		Normoglyce, OGTT, and alloxan-induced diabetic mice	100, 200, and 400	GL	Alkaloids, glycoside, tannins, saponins, Phytosterols, flavonoids, and diterpenes	↓BGL at 200 and 400 mg/kg in normoglycemic mice (*P* < 0.05). ↓BGL (*P* < 0.05) in only 400 mg/kg exposed groups at the 120 mins of postexposure in OGTT model. ↓BGL (*P* < 0.05) at all doses of the extract at 4, 6, and 10 h on diabetic mice.	>2,000	[75]
(17) *Vigna radiata*	Aqueous extract of grain		STZ-induced diabetic mice	200 and 300	GL	Anthocyanin and free and bound phenolic acids	↓BGL at 200 and 300 mg/kg extracts (*P* < 0.05).	ND	[76]
Lamiaceae	Ethanol and aqueous extract of leaf		Alloxan-induced diabetic mice	300 and 500	GL	Steroids, phenols, flavonoids, and tannins	↓Fasting mean BGL in diabetic mice with crude aqueous extract 300 and 500 mg/kg and crude ethanol extract 300 and 500 mg/kg by 27.96, 38.98, 28.09, and 28.25%, respectively (*P* < 0.05).	>5,000	[11]
(18) *Ajuga remota* Benth	Aqueous and 70% ethanol extracts of leaves		Alloxan-induced diabetic mice	300 and 500	GL	Phenols, flavonoids, saponins, tannins, and steroids	↓BGL (*P* < 0.0001) at aqueous extracts 300 and 500 mg/kg by 27.83 ± 2.96% and 38.98 ± 0.67%, respectively. ↓BGL (*P* < 0.05) at 70% ethanol extracts 300 and 500 mg/kg by 27.94 ± 1.92% and 28.26 ± 1.82%, respectively.	>5,000	[77]
	Ethanol extract of leaves		STZ-induced diabetic rats	200 and 400	GL	Diterpenoids, phytoecdysteroids, phenolics, flavonoids, and glycosides	↓Fasting BGL (*P* < 0.05) treated with 200 and 400 mg/kg ethanol extract on 21st and 14th day of treatment, respectively.	ND	[78]
(19) *Becium grandiflorum* Lam.	Ethanolic leaves extract		STZ-induced diabetic mice	200, 400, and 600	GL	Flavonoids terpenoids, tannins, saponins, phenols, and steroids	↓BGL (*P* < 0.001) at 600 mg/kg extract at the 5th day. ↓BGL at all doses of the extract at the end of the 15th day of treatment by 17.61, 22.52, and 24.62%, respectively.	>5,000	[54]
(20) *Otostegia integrifolia* Benth.	80% methanolic leaf extract		Hypoglycemic, OGTT, and STZ-induced diabetic mice	100, 200, and 400	GL	Phenols, saponins, reducing sugars, and flavonoids	↓BGLs at 200 mg/kg extract in the hypoglycemic and OGTT models. ↓Fasting BGL (*P* < 0.001) at 100 and 200 mg/kg doses at 4 h in diabetic mice.	>5,000	[47]
(21) *Thymus schimperi* Ronniger	Methanolic crude leaves extract, *n*-butanol, ethyl acetate, and aqueous fraction of leaves		STZ-induced diabetic mice	250, 500, and 750	GL	Alkaloids, polyphenols, flavonoids, tannins, saponins, and terpenoids.	↓BGL at methanolic crude extract of 250 mg/kg (14.76 ± 6.1%), 500 mg/kg (25.12 ± 11.5%), and 750 mg/kg (27.15 ± 10.0%) in a dose dependent manner. ↓Postprandial hyperglycemia by methanol extract. ↓BGL at a dose of 500 and 250 mg/kg *n*-butanol fraction by 36 ± 7.3% and 22.2 ± 4.3%, respectively. ↓BGL at a dose of aqueous fraction 250 and 500 mg/kg by 17.6% ± 6.0% and 18.4 ± 5.0%, respectively.	>2,000	[46]
	Aqueous and methanolic extracts of leaves		Alloxan-induced diabetic mice	250 and 500	GL	Terpenoids	↓BGL (*P* < 0.05) at 500 mg/kg aqueous and 500 mg/kg 80% methanol extract at 14 and 21 days. ↓BGL (*P* < 0.05) at 500 mg/kg 80% methanol and (*P* < 0.001) at 250 mg/kg aqueous and 80% methanol.	>2,000	[2]
	80% methanol and boiling water extract of leaves	Chromogenic DNSA and yeast *α*-glucosidase enzyme		0.5, 1, 1.5, 2, and 2.5 mg/ml	Acarbose	Phenols and flavonoids	Inhibited *α*-amylase activity (*P* < 0.05) by 68.6 ± 5.9% and 48.7 ± 7.1% at 2.5 mg/mL 80% methanol and boiling water extracts with IC_50_ 0.33 ± 0.05 mg/ml and IC_50_ 2.24 ± 0.53 mg/ml, respectively. Inhibited *α*-glucosidase activity (*p* < 0.05) by 96.8 ± 10.5% and 84.4 ± 8.5% at 2.5 mg/mL hot water and 80% methanol extract with IC_50_ 0.05 ± 0.01 mg/ml and IC_50_ 0.69 ± 0.04 mg/ml, respectively.	ND	[79]
(22) *Thymus vulgaris* L.	80% methanol and boiling water extract of leaves	Chromogenic DNSA and yeast *α*-glucosidase enzyme		0.5, 1, 1.5, 2, and 2.5 mg/ml	Acarbose	Phenols and flavonoids	Inhibited *α*-amylase activity (*P* < 0.05) by 60.7 ± 9.2% and 27.1 ± 3.9% at 80% methanol and hot water extract with IC_50_ 1.56 ± 0.09 mg/ml and IC_50_ > 2.5 mg/ml. Inhibited *α*-glucosidase activity (*P*< 0.05) by 86.7 ± 8.3% and 60.7 ± 9.2% at 2.5 mg/mL hot water and 80% methanol extract with IC_50_ 0.24 ± 0.09 and IC_50_ 0.51 ± 0.02, respectively.	ND	[79]
(23) *Salvia tiliifolia* Vahl	80% methanolic aerial extracts		Alloxan-induced diabetic mice	400	GL	Alkaloid, saponins flavonoids, and phytosterols	↓fasting BGL (*P* < 0.001) at the extract 400 mg/kg at 2, 4, and 6 h.	>2,000	[80]
Malvaceae (24) *Hibiscus deflersii* Schweinf. ex Cufod.	Methanolic extract of leaf		STZ-induced diabetic rats	400	GL	Phytosterols, flavonoids, and glycosides	↓BGL with the continuous treatment of the extract for a period of 15 days.	>5,000	[81]
Meliaceae (25) *Melia azedarach* Linn.	Aqueous leaf extract		Glucose in ob/ob mice	200, 300, and 400	GL	ND	↓BGL at 200, 300, and 400 mg/kg at all the time points (*P* < 0.05–0.001). ↓Parallel fasting BGL (all *P* < 0.001) at 200 and 400 mg/kg in the long-term effects over the 3-week period. ↓insulin levels progressively (*P* < 0.01–0.001) over the 3-week treatment period with 200 or 400 mg/kg.	ND	[28]
Melianthaceae (26) *Bersama abyssinica* Fresen.	Leaves 80% methanol crude extract and water, ethyl acetate, and chloroform fractions	Chromogenic DNSA	Normoglycemic, OGTT, and STZ-induced diabetic mice	100, 200, 400, 50, 100, 200, 400, 600, 800, and 1000 *μ*g/mL	GL and Acarbose	Alkaloids, glycosides, flavonoids, steroids, phenols, tannins, triterpene, anthraquinone, polysterols, and coumarins	↓BGL by 25.59%, 42.60%, and 49.42% for 100, 200, and 400 mg/kg, respectively, in normoglycemic mice. ↓BGL (*P* < 0.05) at all doses of the crude extract in OGTT mice. ↓BGL by 25.71, 33.27, 40.71, and 48.39% at 400 mg/kg chloroform, ethyl acetate and aqueous fraction, and crude extract, respectively, in diabetic mice. Inhibited *α*-amylase with IC_50_ values of crude extract, water fraction, ethyl acetate fraction, and the chloroform fraction 6.57 ± 0.74 *μ*g/mL, 13.33 ± 0.57 *μ*g/mL, 20.34 ± 0.67 *μ*g/mL, and 30.97 ± 0.84 *μ*g/mL, respectively.	>2,000	[37]
*Moringaceae*	Crude aqueous extract, chloroform, and *n*-butanol leaves fraction		Alloxan-induced diabetic mice	500	GL	Alkaloids, saponins, glycoproteins, amino acids, and proteins	↓BGL (*P* < 0.05) for crude aqueous extract at all periods except at 6 h. ↓BGL (*P* < 0.05) for chloroform, *n*-butanol, and aqueous residue fractions at all periods. ↓BGL (*P* < 0.005) for *n*-butanol and aqueous residue fractions at 1.5 h of their administration.	>50.6 g/kg	[82]

(27) *Moringa stenopetala* Baker f.	Ethanol and aqueous extract, petroleum ether, butanol, and chloroform fraction of leaves		Normoglycemic and alloxan-induced diabetic mice	300	GL	ND	↓BGL for ethanol extract at 60, 180, and 240 mins (*P* < 0.01) and 120 mins (*P* < 0.001) in normoglycemic mice. ↓BGL for aqueous and chloroform extracts at 120 (*P* < 0.01), 180 (*P* < 0.05), and 240 mins (*P* < 0.05). ↓BGL (*P* < 0.05) for aqueous crude extract, butanol, and chloroform fraction starting from 180 mins. ↓BGL for ethanol extract at 60 (*P* < 0.05) and 120, 180, and 240 mins (*P* < 0.001). ↓BGL for aqueous extract at 120 mins (*P* < 0.01) and 180 and 240 mins (*P* < 0.001) of single dose in diabetic mice. ↓BGL for the ethanol extract (*P* < 0.001) at 3rd day. ↓BGL for aqueous extract on the 3^rd^ (*P* < 0.01) and 5th and 8th days (*P* < 0.001). ↓BGL for chloroform and butanol fractions on 5th day (*P* < 0.01) and 8th day (*P* < 0.001) in diabetic mice.	>50 g/kg	[83]
	Aqueous, ethanol extract, and *n*-butanol fraction of leaf		STZ-induced diabetic rats	250, 500, and 750	Metformin	Polyphenols, flavonoids, phenols, and flavonoids	↓BGL for aqueous ethanol and *n*-butanol extracts 500 mg/kg (*P* < 0.05) in diabetic rats after 14 days. ↓postprandial BGL for the extracts (*P* < 0.001) at the dose of 750 mg/kg.	ND	[67]
	Maltodextrin (9%) and pectin (1%) leaves extract		STZ-induced diabetic mice models	500, 750, and 1,000	GL	ND	↓BGL for the extracts (*P* < 0.05) with dose dependent manner.	>5,000	[84]
	70% ethanol crude extract and *n*-hexane, dichloromethane, *n*-butanol, and chromatographic fractions (1,2,3,4) of leaves		Alloxan-induced male diabetic mice	500	GL	Flavonoids, phenolic compounds, phenolic, and glycosides	↓BGL (*P* < 0.01) with hexane fraction at 2 and 4 h. ↓BGL (*P* < 0.01) with dichloromethane fractionate after 2, 4, and 6 h. ↓BGL with butanol fraction after 2 and 4 h (*P* < 0.001), with aqueous residue (*P* < 0.01) at 2 and 4 h (*P* < 0.05). ↓BGL (*P* < 0.01) with fraction 2 and 3 after 3 h/ ↓BGL (*P* < 0.05) with fraction 1 at 6 h. ↓BGL (*P* < 0.01) with fraction 4 at 3 h and (*P* < 0.05) at 4, 5, and 6 h.	No toxic reaction for 300 and 600 mg/kg in 13-week subchronic toxicity study	[85]
	Hydroalcoholic extract of leaves	Fructose-induced BSA glycation		0.5, 1, and 2 mg/ml	Aminoguanidine	Polyphenols	Inhibited AGEs formation (*P* < 0.05) by 54.75 ± 0.94% at 2 mg/ml of the extract. ↓concentration of fructosamine. ↓Formation of N^*ε*−^(carboxymethyl) lysine (CML). ↓Amount of amyloid cross *β*-structure.	ND	[86]
Myrtaceae (28) *Myrtus communis* L.	Aqueous and methanolic extracts of leaves		Alloxan-induced diabetic mice	500, 750, and 1000	GL	Polyphinols, tannins, and glucosides	↓BGL for aqueous extract at 500 mg/kg by 61.8% (*P* < 0.003) on 5 h. ↓BGL for methanolic extract by 48% (*P* *<* 0.00003) at 1,000 mg/kg dose level.	>5,000	[87]
(29) *Psidium guajava* L.	Aqueous and ethanol extracts of leaves		Normoglycemic and STZ-induced diabetic mice	250, 500, and 750	GL	Alkaloids, phenols, flavonoids, tannins, and saponins	↓BGL (*P* < 0.05) by 28.76% at 750 mg/kg aqueous extract on 21 day. ↓BGL (*P* < 0.05) at 250 mg/kg aqueous extract on day 21 in diabetic mice. ↓BGL (*P* < 0.05) at 500 mg/kg ethanolic extract at the 2nd week.	>5,000	[88]
Resedaceae (30) *Caylusea abyssinica* (Fresen.)	80% methanolic leaf extract		Induction of diabetes by STZ and OGTT	100, 200, and 300	GL	Saponins, flavonoids, and alkaloids	↓BGL by 100 (*P* < 0.05) and 300 mg/kg extract (*P* < 0.01) starting from the 3rd h, and by 200 mg/kg (*P* < 0.001) as early as the 2nd h in diabetic mice. ↓BGL by 100 mg/kg extract (*P* < 0.01) at 120 mins and 200 mg/kg (*P* < 0.001) at 60 mins in OGTT.	>2,000	[89]
(31) *Caylusea abyssinica* (Fresen.) Fisch. and Mey.	Methanol, chloroform, and aqueous fractions of leaves		Normoglycemic, OGTT, and STZ-induced diabetic rodents	200 and 300	Glibenclamide	Alkaloids, tannins, terpenoids, saponins, steroids, flavonoids, and glycosides	↓BGL by 55.37% (*P* < 0.05) and 59.54% (*P* < 0.001) within 2 h of OGTT by 200 and 300 mg/kg of methanol fraction, respectively. ↓BGL by 80.24% (*P* < 0.001) and 81.05% (*P* < 0.001) with the same fraction and doses, respectively. ↓BGL by 55.33% with 300 mg/kg aqueous fraction.	>2,000	[53]
Rosaceae (32) *Hagenia Abyssinica* (Bruce) J.F.Gmel.	80% methanol flower crude extract and chloroform, water, and ethyl acetate fractions	Chromogenic DNSA		25, 50, 100, 200, 400, and 800 *μ*g/mL	Acarbose	Saponins, tannins, terpenoids, phenols, flavonoids, glycosides, steroids, and anthraquinones	Inhibited *α*-amylase activity by 54.23% with IC_50_ 20.78 *μ*g/mL at 800 *μ*g/mL ethyl acetate fraction. Inhibited *α*–amylase activity with IC_50_ 52.11 ± 0.63, 49.08 ± 0.97 *μ*g/mL, and 28.09 ± 0.75 *μ*g/mL of water and chloroform fraction, and crude extract, respectively.	>2,000	[35]
			STZ-induced diabetic mice	100, 200, and 400	GL		↓BGL (*P* < 0.05) at 400 mg/kg crude extract of single dose and ethyl acetate fraction at 8 h. ↓BGL at 200 mg/kg crude extract and ethyl acetate fraction (*P* < 0.05, on the 7th and 14th days) and crude extract 400 mg/kg (*P* < 0.05 and *P* < 0.01, on the 7th and 14th days, respectively). ↓BGL at 400 mg/kg ethyl acetate fraction (*P* < 0.01 and *P* < 0.001, on the 7th and 14th days, respectively).		
Rubiaceae (33) *Pentas schimperiana* (A. Rich).	Hydroalcoholic and aqueous dried leaf extract and hydroalcoholic fresh leaf extract, chloroform, acetone, and methanol fractionation of leaves		Alloxan-induced diabetic mice	500 and 1,000	GL	Flavonoids, saponins, steroids, and tannins	↓BGL (*P* < .01) from 296.2 to 258.6 mg/dL on 3 h at 500 mg/kg of the fresh leaf hydroalcoholic extract. ↓BGL (*P* < 0.001) from 283.2 to 227.6 mg/dL and from 264.6 to 213 mg/dL on 3 h at 500 mg/kg of each of the hydroalcoholic and the aqueous dried leaf extract, respectively. ↓BGL at a dose of 1,000 mg/kg for fresh leaf hydroalcoholic and dried leaf aqueous extracts by 26.7% (*P* < 0.01) and 26.97% (*P* < 0.001), respectively. ↓BGL with hydroalcoholic dried leaf extract by 19.27% (*P* < 0.001) at 1,000 mg/kg dose on 3 h. ↓BGL with methanol and aqueous at a dose of 500 mg/kg (*P* < 0.001).	>4,000	[14]
(34) *Pentas schimperiana* (A. Rich)	Aqueous dried leaf and hydroalcoholic fresh and dried leaf extracts, aqueous, and methanol fractions		Alloxan-induced diabetic mice	500 and 1,000	GL	Saponins, flavonoids, steroids, and tannins	↓BGL by 26.97%, 21.90%, and 26.70% at 1,000 mg/kg of each of the aqueous dried leaf, hydroalcoholic fresh, and dried leaf extracts, respectively. ↓BGL by 23and 27.2% at a dose of 500 mg/kg the aqueous and methanol fractions prepared from the dried plant material, respectively.	>4,000	[14]
Solanaceae (35) *Capsicum frutescens* L.	Acetone, 80% methanol, water, methanol, and petroleum ether extract of green pepper chili paste	Chromogenic DNSA		2.5 mg/mL	Acarbose	Phenols and flavonoids	Inhibited *α*-amylase activity by 80.10 ± 4.37% at 2.5 mg/mL acetone extracts followed by methanol 80% (63.70 ± 2.53%), water (62.78 ± 4.00%), methanol (58.66 ± 3.50%), and petroleum ether (51.34 ± 4.85%) extracts, respectively (*P* < 0.05).	ND	[90]
(36) *Datura stramonium* Linn.	Hydromethanolic seed extract		STZ-induced diabetic mice	100, 200, and 400	GL	Saponins, alkaloids, flavonoids, phenols, tannins, terpenoids, glycosides, and steroids	↓BGL (*P* < 0.05) at 100 mg/kg (*P* < 0.01) and 200 and 400 mg/kg. ↓BGL (*P* < 0.0l) at all doses of extract on day 7 and 14. ↓BGL (*P* < 0.05) at doses of 200 and 400 mg/kg extract. Improved BW of diabetic mice on day 7 and 14.	>2,000	[43]
	Hydromethanolic root extract		Normoglycemic, OGTT, and STZ-induced diabetic mice	100, 200, and 400	GL	Flavonoids, phenols, tannins, alkaloids, steroids, glycosides, and anthraquinone	↓BGL at doses of 100 mg/kg (*P* < 0.05) and 200 mg/kg (*P* < 0.05) and 400 mg/kg (*P* < 0.01) at 1 and 2 h post OGTT mice. ↓BGL at doses of 100 mg/kg (*P* < 0.05 on day 7 and 14), 200 mg/kg (*P* < 0.05 on day 7 and 14), and 400 mg/kg (*P* < 0.05 on day 7, *P* < 0.01 on day 14) in diabetic mice.	>2,000	[38]
Urticaceae (37) *Urtica simensis* Hocht. ex. A. Rich.	Aqueous, 80% methanolic leaf extract		STZ-induced diabetic mice	100, 200, and 300	GL	ND	↓BGL for the aqueous and methanol extracts by 32.3 and 25.1% (300 mg/kg), respectively, at 3 h (*P* < 0.01). ↓BGL for aqueous and methanol fractions at a dose of 300 mg/kg (*P* < 0.01). ↓BGL for aqueous fraction (*P* < 0.01) at the 3 h (100, 200, and 300 mg/kg).	ND	[20]

*Note.* BGL: blood glucose level; BW: body weight; DNSA: 3,5-dinitrosalicylic acid; GL: glibenclamide; IC_50_: inhibitory concentration; LD_50_: lethal dose 50; ND: result not determined; OGTT: oral glucose tolerance test; *R*_f_: retention factor; and STZ: streptozocin.

## Abbreviations

AGEs: advanced glycation end products
BSA: bovine serum albumin
BGL: blood glucose level
cAMP: cyclic adenosine monophosphate
CML: N*ε*-(carboxymethyl) lysine
DM: diabetes mellitus
GLP-1: glucagon-like peptide 1
GLUT-2: glucose transporter 2
GLUT-4: glucose transporter 4
IC_50_: inhibitory concentration
LD_50_: lethal dose 50
MP: medicinal plants
SGLT-2: sodium-glucose cotransporter 2
STZ: streptozocin
WHO: World Health Organization
